# Administrative healthcare data to predict performance status in lung cancer patients

**DOI:** 10.1016/j.dib.2021.107559

**Published:** 2021-11-11

**Authors:** Anita Andreano, Antonio Giampiero Russo

**Affiliations:** Epidemiology Unit, Agency for Health Protection (ATS) of Milan, C.so Italia 52, Milano 20122, Italy

**Keywords:** Performance status, Lung cancer, Administrative claims, Healthcare, Cancer registry

## Abstract

The dataset includes 4488 patients diagnosed with lung cancer (ICD-O 3[3], C33-C34) between 2010–2012 and 2016–2018 in the territory of the Agency for Health Protection (ATS) of Milan, Italy, and selected from its population cancer registry on the basis of availability of the following information: performance status (PS), age, sex, and stage at diagnosis. The dataset includes also the following variables, extracted from the health databases of the ATS and linked to the variables derived from the cancer registry through deterministic record linkage on a unique key (tax code): Charlson comorbidity index, presence of chronic obstructive pulmonary disease, number of hospitalizations, outpatient visits, emergency accesses and prescribed drugs in the previous year, and dispensed durable medical equipment in the previous three years. The dataset was used to develop a logistic prediction model for PS, dichotomized as ‘poor’ (ECOG, 3–5) and ‘good’ (ECOG, 0–2), on the basis of all other variables in the dataset. The prediction model was developed on a 50% random subsample of the described dataset (development dataset, *n* = 2,244) and validated on the remaining half. The area under the curve (AUC) of the model in the development and validation samples were 0.76 and 0.73, respectively. The developed model was used to predict ‘good’ vs. ‘poor’ PS in a sample of patients with advanced lung cancer, from the same registry and years, for which the information was not available. Researchers using registry data, or electronic claims, to perform studies of oncologic therapy effectiveness for lung cancer could use the reported coefficients to predict PS value, dichotomized as ‘good’ or ‘poor’.

## Specifications Table

**Table:** 

Subject	Oncology
Specific subject area	Evaluation of real-world effectiveness of oncologic therapy for lung cancer
Type of data	Tables
How data were acquired	Data from the Cancer register were extracted from the register database hosted at the Epidemiology Unit of the Agency for Health protection of Milan Variables from the administrative health databases of ATS of Milan, stored in the ATS datawarehouse, were linked to the cancer register data on a unique identifier (tax code). Data were then anonymized. All procedures were performed in the safe environment of the ATS.
Data format	Raw and analyzed data
Parameters for data collection	The dataset includes patients diagnosed with lung cancer (ICD-O 3, C33-C34) between 2010–2012 and 2016–2018 in the territory of the Agency for Health Protection (ATS) of Milan, Italy, and registered in its population cancer registry. Excluded patients were death certificate only, non-malignant and non-epithelial tumours, and patients with missing information on stage or Performance Status.
Description of data collection	Information on patients, including Performance Status, and stage were derived from the cancer registry. All other variables were extracted from the health databases of the Lombardy Regional Health System, available at the ATS level for registered residents, through deterministic record linkage on a unique key (tax code).
Data source location	Agency for Health Protection (ATS) of Milan, Milan, Italy
Data accessibility	The coefficients from the prediction model are included within the article. For ethical reasons access control to the row dataset is required. Data access can be requested by e-mail to epidemiologia@ats-milano.it. A user-specific Data Use Agreement will be set up upon request.
Related research article	A. Andreano, W. Bergamaschi, A.G. Russo, Immune checkpoint inhibitors at any treatment line in advanced NSCLC: Real-world overall survival in a large Italian cohort, Lung Cancer. 159 (2021) 145–152. https://doi.org/10.1016/j.lungcan.2021.06.019.

## Value of the Data

• Performance Status is an important confounding factor in studies of treatment effectiveness for lung cancer. The reported regression coefficients allow prediction of Performance Status in individual lung cancer patients from diverse cohorts, using few variables commonly available from administrative health databases.
• Researchers using registry data, or electronic claims, to perform studies of oncologic therapy effectiveness for lung cancer are frequently confronted with the lack of availability of performance status for a part of or the entire cohort. They could use the reported coefficients to predict Performance Status value, dichotomized as 'good' or 'poor'.
• The dataset and the reported coefficients could be used to perform external validation of the model, including re-calibration for a population with a different baseline risk (e.g. different stage distribution).

## 1 Data Description

The dataset includes 4,488 patients with lung cancer. There are four variables derived from the cancer registry of the Agency for Health Protection (ATS) of Milan: patient Eastern Cooperative Oncology Group (ECOG) performance status (*knownPS:* 0 = Fully active to 5 = Dead), sex (*Sex*: 1 = Male, 2 = Female), age in years (*Age)* and stage (*Stage*: IA, IB, IIA, IIB, IIIA, IIIB, IV), both at the time of diagnosis. The following 7 variables were obtained from the administrative health databases of the ATS: Charlson comorbidity index (*Cindex*, 0 to 11), presence of chronic obstructive pulmonary disease (*COPD*: 1 = yes or 0 = no), number of hospitalizations (*N_admission*), outpatient visits (*N_outpatient_visits*) and emergency access (*N_emergency_access*) in the previous year, dispensed durable medical equipment (Durable_equip: 1 = yes or 0 = no) and number of prescribed drugs (*N_prescription*). The variable *Devel_valid* indicates if the record was randomly assigned to development (D) or validation (V) dataset.

Table 1 displays descriptive statistics of all variables included in the dataset i.e. number and percentages for categorical variables, and median and interquartile range for continuous non-normally distributed variables. Some of the continuous variables are additionally described after categorization, for easier interpretation. The statistics are presented for the entire dataset and separately for: patients with ‘poor’ (ECOG 3-5, *n* = 776) and ‘good’ (ECOG 0-2, *n* = 3712) known performance status; the development (*n* = 2,244) and validation (*n* = 2,244) subsets. The *p-*value for the appropriate test for difference between the development and the validation set (i.e. χ^2^ or Man-Whitney U test) is also presented in the last column of the table.

**Table 1:** Sample characteristics, and number of contacts with the health system and of prescribed drugs in the year prior to lung cancer diagnosis in 4,488 patients with known Performance Status residing in the territory of the Agency for Health Protection (ATS) of Milan.

		Performance status		Random 50% sample used for:		
	Overall *n* = 4,488	Poor (ECOG 3–5) *n* = 776	Good (ECOG 0–2) *n* = 3,712	Development *n* = 2,244	Validation *n* = 2,244	*p-*value
PS at the time of diagnosis ECOG scale (%)						0.044
0	1023 (22.8)			515 (23.0)	508 (22.6)	
1	1855 (41.3)			878 (39.1)	977 (43.5)	
2	834 (18.6)			443 (19.7)	391 (17.4)	
3	436 (9.7)			232 (10.3)	204 (9.1)	
4	177 (3.9)			88 (3.9)	89 (4.0)	
5	163 (3.6)			88 (3.9)	75 (3.3)	
PS at the time of diagnosis = Good (ECOG 0–2) (%)	3712 (82.7)			1836 (81.8)	1876 (83.6)	0.124
Age (median [IQR])	73.00 [66.00, 80.00]	80.00 [72.00, 85.00]	72.00 [65.00, 79.00]	73.00 [66.00, 80.00]	73.00 [65.00, 80.00]	0.086
Sex = Female (%)	1388 (30.9)	223 (28.7)	1165 (31.4)	696 (31.0)	692 (30.8)	0.923
Stage (%)						0.065
IA	470 (10.5)	18 (2.3)	452 (12.2)	242 (10.8)	228 (10.2)	
IB	178 (4.0)	11 (1.4)	167 (4.5)	87 (3.9)	91 (4.1)	
IIA	232 (5.2)	29 (3.7)	203 (5.5)	125 (5.6)	107 (4.8)	
IIB	231 (5.1)	19 (2.4)	212 (5.7)	132 (5.9)	99 (4.4)	
IIIA	728 (16.2)	96 (12.4)	632 (17.0)	377 (16.8)	351 (15.6)	
IIIB	282 (6.3)	39 (5.0)	243 (6.5)	146 (6.5)	136 (6.1)	
IV	2367 (52.7)	564 (72.7)	1803 (48.6)	1135 (50.6)	1232 (54.9)	
Charlson Comorbidity Index (median [IQR])	1.00 [0.00, 2.00]	1.00 [1.00, 2.00]	1.00 [0.00, 2.00]	1.00 [0.00, 2.00]	1.00 [0.00, 2.00]	0.994
Charlson Comorbidity Index (%)						
0	1345 (30.0)	183 (23.6)	1162 (31.3)	688 (30.7)	657 (29.3)	
1	1424 (31.7)	221 (28.5)	1203 (32.4)	729 (32.5)	695 (31.0)	
2	956 (21.3)	188 (24.2)	768 (20.7)	448 (20.0)	508 (22.6)	
≥3	763 (17.0)	184 (23.7)	579 (15.6)	379 (16.9)	384 (17.1)	
Presence of chronic obstructive pulmonary disease (mean (SD))	0.33 (0.47)	0.37 (0.48)	0.32 (0.47)	0.32 (0.47)	0.34 (0.47)	0.465
N of hospital admissions (median [IQR])	0.00 [0.00, 0.00]	0.00 [0.00, 0.00]	0.00 [0.00, 0.00]	0.00 [0.00, 0.00]	0.00 [0.00, 0.00]	0.043
N of hospital admissions (%)						
0	3457 (77.0)	596 (76.8)	2861 (77.1)	1701 (75.8)	1756 (78.3)	
1	685 (15.3)	110 (14.2)	575 (15.5)	353 (15.7)	332 (14.8)	
2	227 (5.1)	45 (5.8)	182 (4.9)	132 (5.9)	95 (4.2)	
≥3	119 (2.7)	25 (3.2)	94 (2.5)	58 (2.6)	61 (2.7)	
N of outpatient visits (median [IQR])	2.00 [0.00, 6.00]	2.00 [0.00, 6.00]	3.00 [0.00, 6.00]	2.00 [0.00, 6.00]	2.00 [0.00, 6.00]	0.477
N of outpatient visits (%)						
0	1275 (28.4)	264 (34.0)	1011 (27.2)	619 (27.6)	656 (29.2)	
1	536 (11.9)	89 (11.5)	447 (12.0)	279 (12.4)	257 (11.5)	
2–5	1403 (31.3)	199 (25.6)	1204 (32.4)	714 (31.8)	689 (30.7)	
6–10	766 (17.1)	133 (17.1)	633 (17.1)	368 (16.4)	398 (17.7)	
>10	508 (11.3)	91 (11.7)	417 (11.2)	264 (11.8)	244 (10.9)	
N of emergency department admissions (median [IQR])	0.00 [0.00, 0.00]	0.00 [0.00, 0.00]	0.00 [0.00, 0.00]	0.00 [0.00, 0.00]	0.00 [0.00, 0.00]	0.631
N of emergency department admissions (%)						
0	3918 (87.3)	677 (87.2)	3241 (87.3)	1954 (87.1)	1964 (87.5)	
1	348 (7.8)	51 (6.6)	297 (8.0)	174 (7.8)	174 (7.8)	
2	122 (2.7)	18 (2.3)	104 (2.8)	62 (2.8)	60 (2.7)	
≥3	100 (2.2)	30 (3.9)	70 (1.9)	54 (2.4)	46 (2.0)	
Dispensed durable medical equipment = Yes (%)	33 (0.7)	13 (1.7)	20 (0.5)	16 (0.7)	17 (0.8)	1
N of prescribed drugs (median [IQR])	5.00 [2.00, 8.00]	6.00 [3.00, 9.00]	5.00 [2.00, 8.00]	5.00 [2.00, 8.00]	5.00 [2.00, 8.00]	0.701
N of prescribed drugs (%)						
0	520 (11.6)	100 (12.9)	420 (11.3)	257 (11.5)	263 (11.7)	
1	294 (6.6)	27 (3.5)	267 (7.2)	132 (5.9)	162 (7.2)	
2–5	1580 (35.2)	224 (28.9)	1356 (36.5)	799 (35.6)	781 (34.8)	
6–10	1553 (34.6)	294 (37.9)	1259 (33.9)	788 (35.1)	765 (34.1)	
>10	541 (12.1)	131 (16.9)	410 (11.0)	268 (11.9)	273 (12.2)	

Table 2 displays the estimated logistic regression parameters (β) and standard srrors (s.e.) for the model predicting ‘good’ Performance Status (ECOG 0-2) in lung cancer patients using cancer registry data and information derived from administrative databases of the ATS of Milan.

**Table 2:** Estimated Logistic Regression Parameters (β) and Standard Errors (s.e.) for the model predicting 'good' Performance Status (ECOG 0–2) in lung cancer patients using cancer registry data and information derived from administrative databases of the Agency for Health Protection (ATS) of Milan.

Variables		β	s.e.	*p-*value
	Model Intercept	6.1081	1.835	0.0009
Age (years)	age*	−0.022	0.0269	0.414
	age1*	−0.06	0.0259	0.0204
Sex (Male vs. female)		−0.3723	2.1813	0.8645
Stage (vs. IA)	IB	−1.0631	0.6173	0.0851
	IIA	−1.4593	0.5642	0.0097
	IIB	−0.9154	0.5866	0.1186
	IIIA	−1.7696	0.4823	0.0002
	IIIB	−1.5599	0.5343	0.0035
	IV	−2.5431	0.4643	<0.0001
Charlson Comorbidity Index		0.0079	0.0963	0.9343
Chronic obstructive pulmonary disease (yes vs. no)		−0.1336	0.1429	0.3499
N of hospital admissions		0.0227	0.0826	0.7831
N of outpatient visits		0.0363	0.0137	0.0081
N of emergency department admissions		0.087	0.0984	0.3767
Dispensed durable medical equipment (yes vs. no)		−1.0221	0.5605	0.0682
N of prescribed drugs		−0.0236	0.0247	0.3388
Interactions	age* x sex	0.0021	0.0328	0.9498
	age1* x sex	0.0095	0.0316	0.7647
	Charlson Comorbidity Index x N of prescribed drugs	−0.0124	0.0094	0.1856

⁎ Restricted cubic spline basis functions for age, 3 knots.

## 2 Experimental Design, Materials and Methods

The scale of Performance Status (PS), developed by the Eastern Cooperative Oncology Group (ECOG) in 1982 [1] describes patient's level of functioning in terms of their ability to care for themself, daily activity, and physical ability (walking, working, etc.). We wanted to estimate the average treatment effect (ATE) of immune checkpoint inhibitors in any line of treatment in a 2016–2018 population-based cohort of patients with advanced non-small cell lung cancer (NSCLC) [2]. PS was among the variables needed for adjustment, but it was available only in 23% of the 1673 patients included in the study. To obtain the information for the remaining patients, a prediction model for PS dichotomized as ‘good’ or ‘poor’ was then developed on the presently described dataset.

The dataset includes all patients diagnosed with lung cancer (ICD-O 3[3], C33-C34) between 2010–2012 and 2016–2018 in the territory of the Agency for Health Protection (ATS) of Milan, Italy, and registered in its population cancer registry, member of the International Association of Cancer Registries (IACR) [4]. The number of incident cases of lung tumour in the period was 14,441. Excluded patients were death certificate only (DCO, i.e. diagnosed only by means of death certificate, *n* = 311), non-malignant tumours (ICD-O-3 [3] behaviour code different from /3, *n* = 67), non-epithelial tumours (morphology ICD-O-3 [3] code equal or higher than 8680/3, *n* = 63) and patients with missing information on stage (*n* = 2,767). Of the remaining 11,233 patients, 4,488 had a recorded PS value in the cancer registry, either abstracted from clinical records or derived from trained research nurses from the same source, and were used to develop (*n* = 2,244 random records) and validate (*n* = 2,244 remaining records) the prediction model.

Age in years at diagnosis, sex, and TNM 8th edition stage at diagnosis [5] were derived from the cancer registry. The remaining variables were derived from administrative health databases of the Lombardy Regional Health System, available at the ATS level for registered residents, as following:

‐ number of hospitalizations in the year before diagnosis: by tax code, sum of any hospital admission recorded in the hospital discharge sheet (SDO) database in the 365 days starting 30 days before date of diagnosis.
‐ number of outpatient visits in the year before diagnosis: by tax code, sum of any record with ICD-9-CM code starting with ‘89’ in the outpatient database in the 365 days starting 30 days before date of diagnosis.
‐ number of emergency accesses in the year before diagnosis: by tax code, sum of any emergency room access recorded in the emergency care database in the 365 days starting 30 days before date of diagnosis.
‐ dispensed durable medical equipment in the 3 years before diagnosis: by tax code, if a durable medical equipment among portable oxygen, walkers, canes, wheelchairs, and hospital beds had been dispensed in the 365*3 days starting 30 days before date of diagnosis.
‐ number of prescribed drugs in the year before diagnosis: by tax code, number of prescribed drugs with different ATC codes [6] in the outpatient drug dataset in the 365 days starting 30 days before date of diagnosis.
‐ Charlson comorbidity index was calculated adapting the algorithm from Quan et al. [7], based on hospital discharge sheets, to include information from the outpatient drug and exemption from co-payment datasets, the latter including exemptions for named chronic diseases. The specification of the algorithm for defining presence of the different chronic diseases included in the comorbidity index from the outpatient drug and exemption from co-payment datasets are the same described in supplementary material of Murtas et al. [8].
‐ chronic obstructive pulmonary disease was considered as present if the subject had an hospitalization prior to diagnosis with the following ICD-9-CM codes in any diagnosis field: 416.8, 416.9, 490.x to 505.x, 506.4, 508.1, 508.8 [7]; or if he had more than 45 years and prescription of drugs starting with ATC code R03 (drugs for obstructive airway diseases) and a defined daily dose (DDD) [6] of at least 30% in the year before diagnosis.

Date of diagnosis in the above calculations was always the date recorded in the cancer register which, as per international rules, is the date of first histological or cytological confirmation when available [9]. For this reason, the events in the 30 days preceding this date of diagnosis were not counted, as likely related to the lung cancer diagnosis process. Deterministic record linkage on a unique key was used to match all information at patient level within the information system of the ATS, which houses the cancer registry and the administrative data, and was anonymized prior to analysis. The resulting dataset is described in Table 1.

A logistic regression model was fitted on a 50% random subsample of the described dataset (development dataset, *n* = 2,244) with dichotomized PS ‘Good’ (ECOG 0–2) vs. ‘Poor’ (ECOG 3–5) as the dependent variable and the following predictors: age, sex, stage, Charlson's index, chronic obstructive pulmonary disease, number of hospital admissions, outpatient visits, emergency accesses and prescribed drugs in the previous year, and durable medical equipment in the previous three years. The predictors to be included in the model were chosen on the basis of the literature [5]. It was decided to develop a full model, without performing model selection using automatic statistical techniques, given the high number of events and the minimal cost represented by the collection of this information, and in order to maximize the expected discrimination ability based on administrative data only. The interactions included in the model (age x sex, and Charlson Comorbidity Index x N of prescribed drugs) were pre-specified on subject-matter knowledge basis. Age was included in the model as a restricted cubic spline with 3 knots. Coefficients and standard errors for the fitted model are presented in Table 2. The AUC of the model in the development sample was 0.76, the Brier score 0.12. The AUC of the developed model on the validation dataset was 0.73, the Brier score 0.12. Intercept and calibration slope in the validation dataset were 0.27 and 0.81.

## Ethics Statement

The study project of which these data are a part has been approved from ethics committee Milan Area 2 (protocol review number 231_2021bis of March 17 2021, id study 2059).

## CRediT authorship contribution statement

**Anita Andreano:** Conceptualization, Methodology, Formal analysis, Investigation, Writing – original draft, Writing – review & editing. **Antonio Giampiero Russo:** Conceptualization, Methodology, Supervision, Writing – review & editing.

## Declaration of Competing Interest

These data, and the described analysis, have been used within a project supported by Roche S.p.A. e M.S.D. Italia s.r.l. The funder had no role in the study design, data collection and analysis, decision to publish, or preparation of the manuscript. The authors declare that they have no other competing financial interests or personal relationships which have or could be perceived to have influenced the work reported in this article.
